# Supplementary material for: Direct comparisons of neural activity during placebo analgesia and nocebo hyperalgesia between humans and rats
Source: Commun Biol. 2025 Apr 5;8:570. doi: 10.1038/s42003-025-07993-1 (PMC11972415; doi:10.1038/s42003-025-07993-1)
Supplement: Supplementary file 1 — Supplementary Information [file 42003_2025_7993_MOESM1_ESM.pdf]

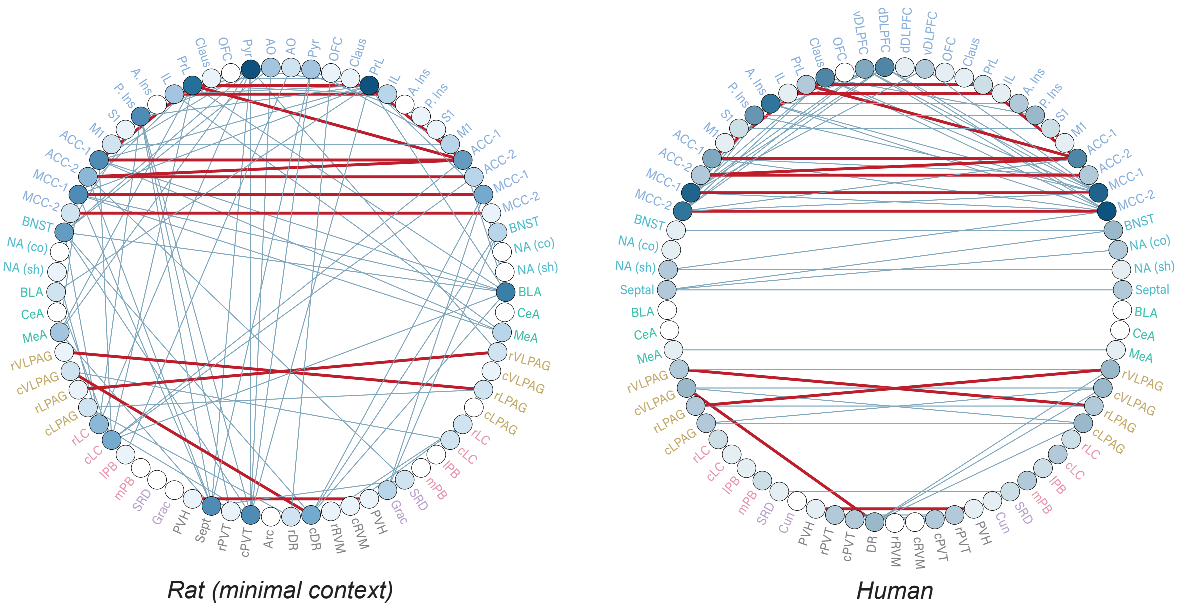

Supplementary Figure 1: Functional connectivity maps for control subjects (moderate pain control test or scan). Functional connectivity maps between all ROIs were first generated using c-Fos expression in rats and beta-values in humans, with the top 100 strongest connections for each group identified, and circos graphs were then created using Gephi. The darkness of the nodes reflect the degree of connectivity in the network. Red lines indicate shared connections between rats and humans. AO, anterior olfactory tubercle, Pyr, pyriform cortex, aINS, anterior insula, pINS, posterior insula cortex, PrL, prelimbic cortex, IL, infralimbic cortex, OFC, orbitofrontal cortex, ACC_1, dorsal anterior cingulate cortex, ACC_2, ventral anterior cingulate cortex, Cl, claustrum, Sept, septal nuclei, BNST, bed nucleus of the stria terminalis, NAc, nucleus accumbens core, NaSh, nucleus accumbens shell, M1, primary motor cortex, S1, primary somatosensory cortex, PVT, paraventricular thalamus, PVH, paraventricular hypothalamus, CeA, central nucleus of the amygdala, MeA, medial nucleus of the amygdala, BLA, basolateral nucleus of the amygdala, lPAG, lateral periaqueductal gray, vlPAG, ventrolateral periaqueductal gray, DR, dorsal raphe, LC, locus coeruleus, mPB, medial parabrachial nucleus, lPB, lateral parabrachial nucleus, RVM, rostral ventromedial medulla, SRD, subnucleus reticularis dorsalis, Gr, gracile nucleus, ACC, anterior cingulate cortex, MCC_1, dorsal middle cingulate cortex, MCC_2, ventral middle cingulate cortex, Cun, cuneate nucleus.

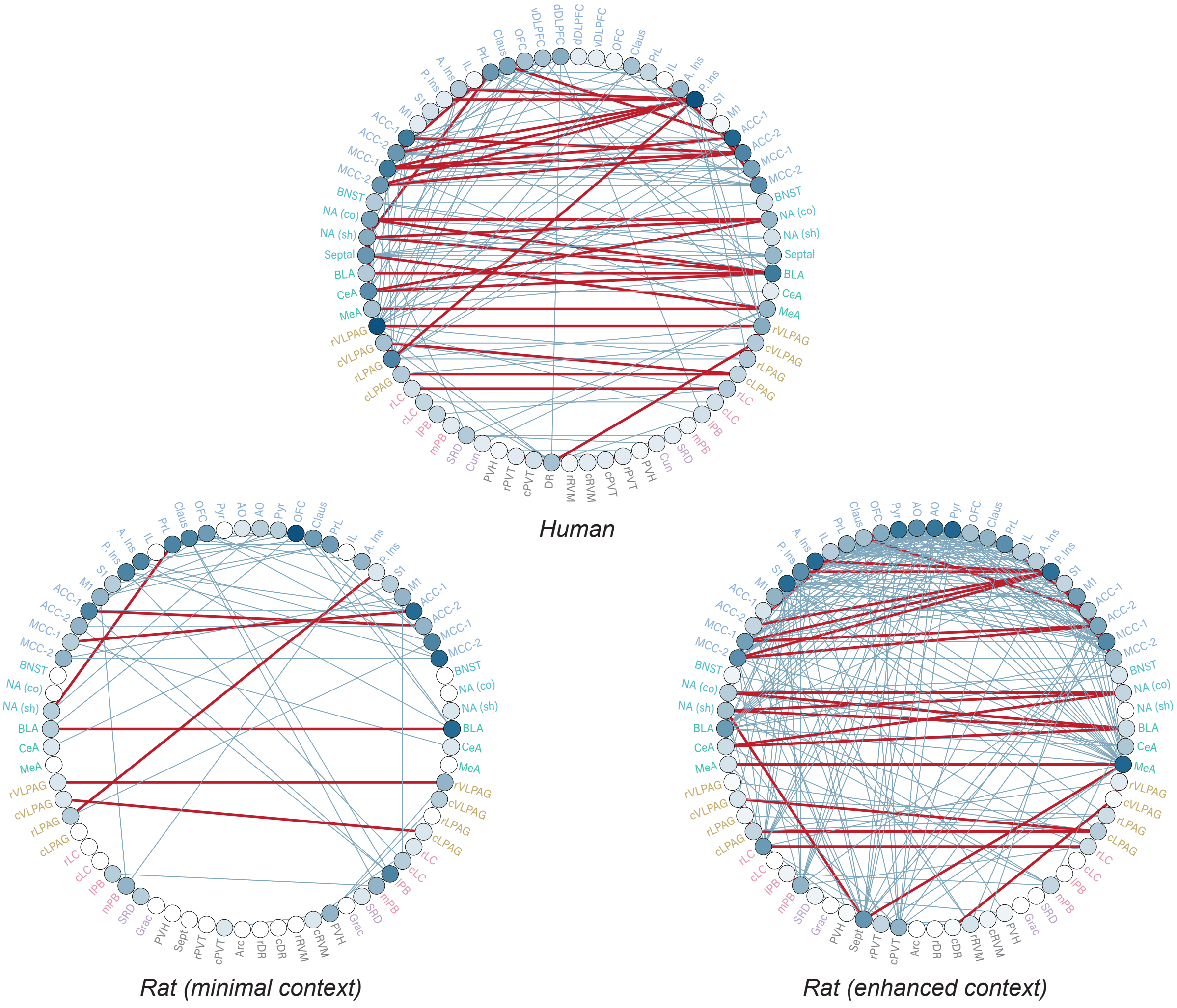

Supplementary Figure 2: Functional connectivity maps during placebo analgesia. Functional connectivity maps between all ROIs were first generated using c-Fos expression in rats and beta-values in humans. The number of connections shown for each group is relative to controls, with the same r-value cut-offs applied as used in the control groups (rats: r =0.879, humans: r =0.699), and circos graphs were then created using Gephi. The darkness of the nodes reflect the degree of connectivity in the network. Red lines indicate shared connections between rats and humans. AO, anterior olfactory tubercle, Pyr, pyriform cortex, aINS, anterior insula, pINS, posterior insula cortex, PrL, prelimbic cortex, IL, infralimbic cortex, OFC, orbitofrontal cortex, ACC_1, dorsal anterior cingulate cortex, ACC_2, ventral anterior cingulate cortex, Cl, claustrum, Sept, septal nuclei, BNST, bed nucleus of the stria terminalis, NAc, nucleus accumbens core, NaSh, nucleus accumbens shell, M1, primary motor cortex, S1, primary somatosensory cortex, PVT, paraventricular thalamus, PVH, paraventricular hypothalamus, CeA, central nucleus of the amygdala, MeA, medial nucleus of the amygdala, BLA, basolateral nucleus of the amygdala, lPAG, lateral periaqueductal gray, vlPAG, ventrolateral periaqueductal gray, DR, dorsal raphe, LC, locus coeruleus, mPB, medial parabrachial nucleus, lPB, lateral parabrachial nucleus, RVM, rostral ventromedial medulla, SRD, subnucleus reticularis dorsalis, Gr, gracile nucleus, ACC, anterior cingulate cortex, MCC_1, dorsal middle cingulate cortex, MCC_2, ventral middle cingulate cortex, Cun, cuneate nucleus.

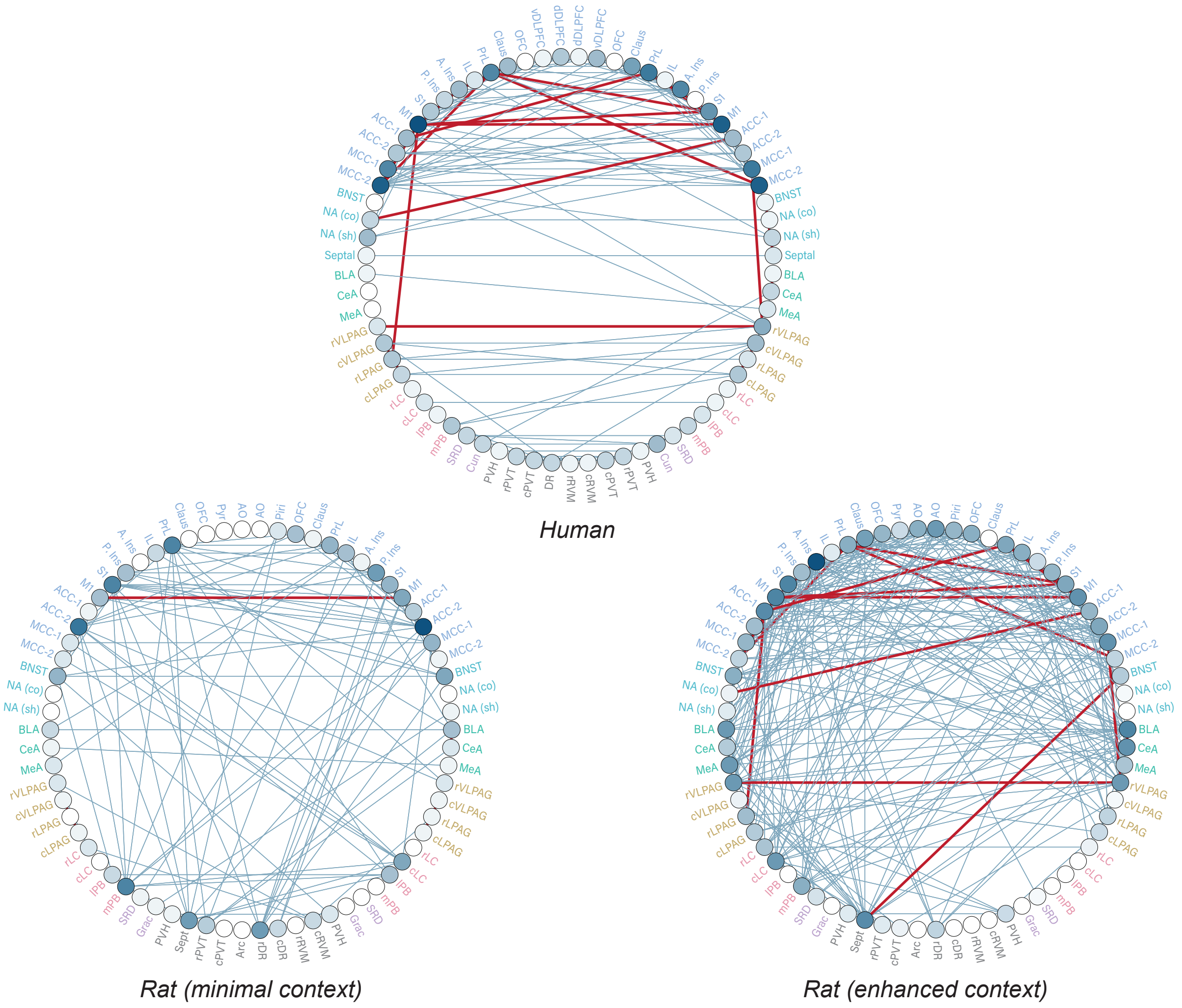

Supplementary Figure 3: Functional connectivity maps during nocebo hyperalgesia. Functional connectivity maps between all ROIs were first generated using c-Fos expression in rats and beta-values in humans. The number of connections shown for each group is relative to controls, with the same r-value cut-offs applied as used in the control groups (rats: r =0.879, humans: r =0.699), and circos graphs were then created using Gephi. The darkness of the nodes reflect the degree of connectivity in the network. Red lines indicate shared connections between rats and humans. AO, anterior olfactory tubercle, Pyr, pyriform cortex, aINS, anterior insula, pINS, posterior insula cortex, PrL, prelimbic cortex, IL, infralimbic cortex, OFC, orbitofrontal cortex, ACC_1, dorsal anterior cingulate cortex, ACC_2, ventral anterior cingulate cortex, Cl, claustrum, Sept, septal nuclei, BNST, bed nucleus of the stria terminalis, NAc, nucleus accumbens core, NaSh, nucleus accumbens shell, M1, primary motor cortex, S1, primary somatosensory cortex, PVT, paraventricular thalamus, PVH, paraventricular hypothalamus, CeA, central nucleus of the amygdala, MeA, medial nucleus of the amygdala, BLA, basolateral nucleus of the amygdala, lPAG, lateral periaqueductal gray, vlPAG, ventrolateral periaqueductal gray, DR, dorsal raphe, LC, locus coeruleus, mPB, medial parabrachial nucleus, lPB, lateral parabrachial nucleus, RVM, rostral ventromedial medulla, SRD, subnucleus reticularis dorsalis, Gr, gracile nucleus, ACC, anterior cingulate cortex, MCC_1, dorsal middle cingulate cortex, MCC_2, ventral middle cingulate cortex, Cun, cuneate nucleus.

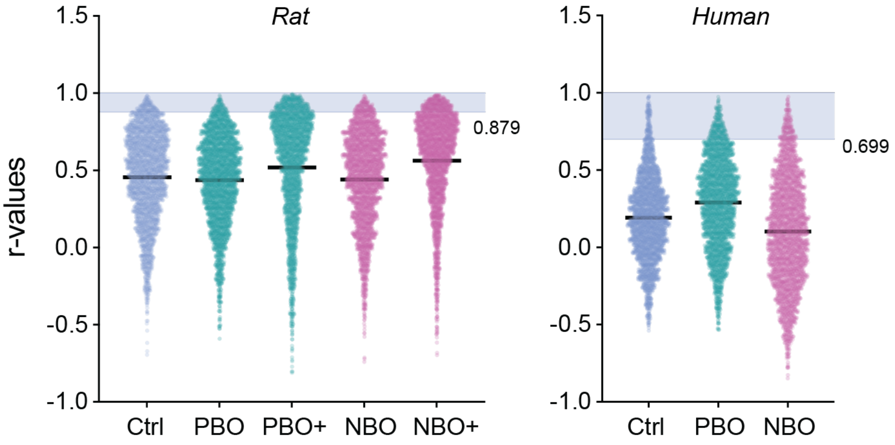

Supplementary Figure 4. Distribution of Pearson's r-values for functional connectivity analysis for each group. Blue shaded region indicates the values included based on the cut-off r-value for the top 100 connections in each of the control groups. Black line indicates the mean. Ctrl, control, PBO, placebo, NBO, nocebo, PBO+ placebo enhanced context, NBO+, nocebo enhanced context.

Supplementary Table 1: Demographic data and measures of pain expectations for each cream prior to fMRI scans. Two-way, repeated measures ANOVAs with post-hoc multiple comparisons (Tukey correction) were used to compare expectations between creams, and between responders and non-responders. Unpaired t-tests were used to compare the age between responders and non-responders, and Fisher's exact test was used to compare distributions of sexes between responders and non-responders.

**Demographics (Placebo)**	**Age**	**Sex**	
Placebo Responder (n=22)	23.95±0.71	14M / 8F	
Placebo Non-responder (n=24)	24.04±0.94	10M / 15F	
Responder vs. Non-responder	p=0.94	p=0.15	
**Demographics (Nocebo)**	**Age**	**Sex**	
Nocebo Responder (n=14)	22.29±0.91	8M / 6F	
Nocebo Non-responder (n=11)	23.27±1.09	4M 7F	
Responder vs. Non-responder	p=0.50	p=0.43	
**Expectation (Placebo)**	**Vaseline**	**Lidocaine**	**Vaseline vs. Lidocaine**
Placebo Responder (n=22)	49.35±0.77	33.48±1.63	p<0.0001
Placebo Non-responder (n=24)	51.67±1.76	37.14±2.71	p<0.0001
Responder vs. Non-responder	p=0.82	p=0.52	
**Expectation (Nocebo)**	**Vaseline**	**Capsaicin**	**Vaseline vs. Capsaicin**
Nocebo Responder (n=14)	48.33±0.90	68.33±2.85	p<0.0001
Nocebo Non-responder (n=11)	48.00±1.90	62.50±3.10	p=0.0008
Responder vs. Non-responder	p=0.99	p=0.30	

Supplementary Table 2: List of all ROIs in rats and humans and abbreviations. Regions are aligned with their homologous structures in each species.

Rat Full Name	Rat ROI code	Human Full Name	Human ROI code
Anterior Cingulate Cortex CG1 Left	ACC_1-L	Anterior Cingulate Cortex CG1 Left	ACC_1-L
Anterior Cingulate Cortex CG1 Right	ACC_1-R	Anterior Cingulate Cortex CG1 Right	ACC_1-R
Anterior Cingulate Cortex CG2 Left	ACC_2-L	Anterior Cingulate Cortex CG2 Left	ACC_2-L
Anterior Cingulate Cortex CG2 Right	ACC_2-R	Anterior Cingulate Cortex CG2 Right	ACC_2-R
Anterior Insula Left	aINS-L	Anterior Insula Left	aINS-L
Anterior Insula Right	aINS-R	Anterior Insula Right	aINS-R
Anterior Olfactory Nucleus Left	AO-L		
Anterior Olfactory Nucleus Right	AO-R		
Arcuate Nucleus	Arc		
Basolateral Amygdala Left	BLA-L	Basolateral Amygdala Left	BLA-L
Basolateral Amygdala Right	BLA-R	Basolateral Amygdala Right	BLA-R
Bed Nucleus of the Stria Terminalis Left	BNST-L	Bed Nucleus of the Stria Terminalis Left	BNST-L
Bed Nucleus of the Stria Terminalis Right	BNST-R	Bed Nucleus of the Stria Terminalis Right	BNST-R
Caudal Dorsal Raphe	cDR	Dorsal Raphe	DR
Caudal Lateral Periaqueductal Gray Left	cLPAG-L	Caudal Lateral Periaqueductal Gray Left	cLPAG-L
Caudal Lateral Periaqueductal Gray Right	cLPAG-R	Caudal Lateral Periaqueductal Gray Right	cLPAG-R
Caudal Locus Coeruleus Left	cLC-L	Caudal Locus Coeruleus Left	cLC-L
Caudal Locus Coeruleus Right	cLC-R	Caudal Locus Coeruleus Right	cLC-R
Caudal Paraventricular Thalamus	cPVT	Caudal Paraventricular Thalamus Left	cPVT-L
		Caudal Paraventricular Thalamus Right	cPVT-R
Caudal Rostral Ventromedial Medulla	cRVM	Caudal Rostral Ventromedial Medulla	cRVM
Caudal Ventrolateral Periaqueductal Gray Left	cVLPAG-L	Caudal Ventrolateral Periaqueductal Gray Left	cVLPAG-L
Caudal Ventrolateral Periaqueductal Gray Right	cVLPAG-R	Caudal Ventrolateral Periaqueductal Gray Right	cVLPAG-R
Central Amygdala Left	CeA-L	Central Amygdala Left	CeA-L
Central Amygdala Right	CeA-R	Central Amygdala Right	CeA-R
Claustrum Left	Claus-L	Claustrum Left	Claus-L
Claustrum Right	Claus-R	Claustrum Right	Claus-R
Core Nucleus Accumbens Left	NaCo-L	Core Nucleus Accumbens Left	NaCo-L
Core Nucleus Accumbens Right	NaCo-R	Core Nucleus Accumbens Right	NaCo-R
Gracile Nucleus Left	Grac-L	Cuneate Nucleus Left	Cun-L
Gracile Nucleus Right	Grac-R	Cuneate Nucleus Right	Cun-R
		Dorsolateral Prefrontal Cortex Dorsal Left	dDLPFC-L
		Dorsolateral Prefrontal Cortex Dorsal Right	dDLPFC-R
		Dorsolateral Prefrontal Cortex Ventral Left	vDLPFC-L
		Dorsolateral Prefrontal Cortex Ventral Right	vDLPFC-R
Infralimbic Cortex Left	IL-L	Infralimbic Cortex Left	IL-L
Infralimbic Cortex Right	IL-R	Infralimbic Cortex Right	IL-R
Lateral Parabrachial Nucleus Left	PBl-L	Lateral Parabrachial Nucleus Left	PBl-L
Lateral Parabrachial Nucleus Right	PBl-R	Lateral Parabrachial Nucleus Right	PBl-R
Medial Amygdala Left	MeA-L	Medial Amygdala Left	MeA-L
Medial Amygdala Right	MeA-R	Medial Amygdala Right	MeA-R
Medial Parabrachial Nucleus Left	PBm-L	Medial Parabrachial Nucleus Left	PBm-L
Medial Parabrachial Nucleus Right	PBm-R	Medial Parabrachial Nucleus Right	PBm-R
Midcingulate Cortex CG1 Left	MCC_1-L	Midcingulate Cortex CG1 Left	MCC_1-L
Midcingulate Cortex CG1 Right	MCC_1-R	Midcingulate Cortex CG1 Right	MCC_1-R
Midcingulate Cortex CG2 Left	MCC_2-L	Midcingulate Cortex CG2 Left	MCC_2-L
Midcingulate Cortex CG2 Right	MCC_2-R	Midcingulate Cortex CG2 Right	MCC_2-R
Orbitofrontal Cortex Left	OFC-L	Orbitofrontal Cortex Left	OFC-L
Orbitofrontal Cortex Right	OFC-R	Orbitofrontal Cortex Right	OFC-R
Paraventricular Hypothalamus Left	PVH-L	Paraventricular Hypothalamus Left	PVH-L
Paraventricular Hypothalamus Right	PVH-R	Paraventricular Hypothalamus Right	PVH-R
Piriform Cortex Left	Pyr-L		
Piriform Cortex Right	Pyr-R		
Posterior Insula Left	pINS-L	Posterior Insula Left	pINS-L
Posterior Insula Right	pINS-R	Posterior Insula Right	pINS-R
Prelimbic Cortex Left	PrL-L	Prelimbic Cortex Left	PrL-L
Prelimbic Cortex Right	PrL-R	Prelimbic Cortex Right	PrL-R
Primary Motor Cortex Left	M-L	Primary Motor Cortex Left	M-L
Primary Motor Cortex Right	M-R	Primary Motor Cortex Right	M-R
Primary Somatosensory Cortex Left	S1-L	Primary Somatosensory Cortex Left	S1-L
Primary Somatosensory Cortex Right	S1-R	Primary Somatosensory Cortex Right	S1-R
Rostral Dorsal Raphe	rDR	Dorsal Raphe	DR
Rostral Lateral Periaqueductal Gray Left	rLPAG-L	Rostral Lateral Periaqueductal Gray Left	rLPAG-L
Rostral Lateral Periaqueductal Gray Right	rLPAG-R	Rostral Lateral Periaqueductal Gray Right	rLPAG-R
Rostral Locus Coeruleus Left	rLC-L	Rostral Locus Coeruleus Left	rLC-L
Rostral Locus Coeruleus Right	rLC-R	Rostral Locus Coeruleus Right	rLC-R
Rostral Paraventricular Thalamus	rPVT	Rostral Paraventricular Thalamus Left	rPVT-L
		Rostral Paraventricular Thalamus Right	rPVT-R
Rostral Rostral Ventromedial Medulla	rRVM	Rostral Rostral Ventromedial Medulla	rRVM
Rostral Ventrolateral Periaqueductal Gray Left	rVLPAG-L	Rostral Ventrolateral Periaqueductal Gray Left	rVLPAG-L
Rostral Ventrolateral Periaqueductal Gray Right	rVLPAG-R	Rostral Ventrolateral Periaqueductal Gray Right	rVLPAG-R
Septal Nucleus	Sept	Septal Nucleus Left	Sept-L
		Septal Nucleus Right	Sept-R
Shell Nucleus Accumbens Left	NaSh-L	Shell Nucleus Accumbens Left	NaSh-L
Shell Nucleus Accumbens Right	NaSh-R	Shell Nucleus Accumbens Right	NaSh-R
Subnucleus Reticularis Dorsalis Left	SRD-L	Subnucleus Reticularis Dorsalis Left	SRD-L
Subnucleus Reticularis Dorsalis Right	SRD-R	Subnucleus Reticularis Dorsalis Right	SRD-R

Supplementary Table 3: Functional connectivity network metrics (Supplementary Figures 1-3). Metrics were computed by Gephi software.

Group	Graph density	Network diameter	Average clustering coefficient	Average degree	Avg path length
Rat control	0.080	9	0.379	5.714	3.145
Rat PBO min	0.057	11	0.390	3.943	4.373
Rat PBO enh	0.241	5	0.556	16.629	2.480
Rat NBO min	0.087	6	0.635	6.000	2.384
Rat NBO enh	0.222	8	0.570	15.314	2.621
Human control	0.080	5	0.661	5.634	2.392
Human PBO	0.161	9	0.519	11.26	3.479
Human NBO	0.105	6	0.567	7.324	2.502

Graph density: Measures how close the network is to complete. A complete graph has all possible edges and density equal to 1.
Network diameter: The diameter is the longest graph distance between any two nodes in the network (i.e., how far apart are the two most distant nodes).
Average clustering coefficient: The clustering coefficient indicates how nodes are embedded in their neighbourhood. The average gives an overall indication of the clustering in the network.
Average degree: The average number of connections per node.
Average path length: The average graph-distance between all pairs of nodes. Connected nodes have a graph distance of 1.
