# Supplementary material for: Direct comparisons of neural activity during placebo analgesia and nocebo hyperalgesia between humans and rats
Source: Commun Biol. 2025 Apr 5;8:570. doi: 10.1038/s42003-025-07993-1 (PMC11972415; doi:10.1038/s42003-025-07993-1)
Supplement: Supplementary file 2 — Description of Additional Supplementary Materials [file 42003_2025_7993_MOESM2_ESM.pdf]

Description of Additional Supplementary Files

File name: Supplementary Data 1

Description: The source data behind all graphs in the paper

File name: Supplementary Data 2

Description: Results from Estimation Statistics for all ROIs for rats and humans. *indicates the ROIs without equivalent in the other species.
