# Supplementary material for: Direct comparisons of neural activity during placebo analgesia and nocebo hyperalgesia between humans and rats
Source: Commun Biol. 2025 Apr 5;8:570. doi: 10.1038/s42003-025-07993-1 (PMC11972415; doi:10.1038/s42003-025-07993-1)
Supplement: Supplementary file 5 — Reporting summary [file 42003_2025_7993_MOESM5_ESM.pdf]

nature portfolio

Corresponding author(s): Damien Boorman

Last updated by author(s): Mar 15, 2025

Reporting Summary

Nature Portfolio wishes to improve the reproducibility of the work that we publish. This form provides structure for consistency and transparency in reporting. For further information on Nature Portfolio policies, see our Editorial Policies and the Editorial Policy Checklist.

Statistics

For all statistical analyses, confirm that the following items are present in the figure legend, table legend, main text, or Methods section.

n/a	Confirmed	
☐	☒	The exact sample size (*n*) for each experimental group/condition, given as a discrete number and unit of measurement
☐	☒	A statement on whether measurements were taken from distinct samples or whether the same sample was measured repeatedly
☐	☒	The statistical test(s) used AND whether they are one- or two-sided<br>*Only common tests should be described solely by name; describe more complex techniques in the Methods section.*
☒	☐	A description of all covariates tested
☐	☒	A description of any assumptions or corrections, such as tests of normality and adjustment for multiple comparisons
☐	☒	A full description of the statistical parameters including central tendency (e.g. means) or other basic estimates (e.g. regression coefficient) AND variation (e.g. standard deviation) or associated estimates of uncertainty (e.g. confidence intervals)
☒	☐	For null hypothesis testing, the test statistic (e.g. *F*, *t*, *r*) with confidence intervals, effect sizes, degrees of freedom and *P* value noted<br>*Give P values as exact values whenever suitable.*
☒	☐	For Bayesian analysis, information on the choice of priors and Markov chain Monte Carlo settings
☒	☐	For hierarchical and complex designs, identification of the appropriate level for tests and full reporting of outcomes
☐	☒	Estimates of effect sizes (e.g. Cohen's *d*, Pearson's *r*), indicating how they were calculated

Our web collection on statistics for biologists contains articles on many of the points above.

Software and code

Policy information about availability of computer code

Data collection	Functional magnetic resonance imaging (fMRI) sequences were acquired using a whole-body Siemens MAGNETOM 7 Tesla (7T) MRI system (Siemens Healthcare, Erlangen, Germany) with a combined single-channel transmit and 32-channel receive head coil (Nova Medical, Wilmington MA, USA).

Data analysis	MRI Image preprocessing and statistical analyses were performed using Statistical Parametric Mapping (SPM12) and custom software (Diedrichsen, J (2006), 'A spatially unbiased atlas template of the human cerebellum', Neuroimage, vol. 33, no. 1, pp. 127-38.). Images were linearly detrended to remove global signal changes, physiological noise relating to cardiac and respiratory frequency was removed using the DRIFTER toolbox (Sarkka, S, Solin, A, Nummenmaa, A, Vehtari, A, Auranen, T, Vanni, S & Lin, FH (2012), 'Dynamic retrospective filtering of physiological noise in BOLD fMRI: DRIFTER', Neuroimage, vol. 60, no. 2, pp. 1517-27), and the 6-parameter movement related signal changes were modelled and removed using a linear modelling of realignment parameters procedure (Macey, PM, Macey, KE, Kumar, R & Harper, RM (2004), 'A method for removal of global effects from fMRI time series', Neuroimage, vol. 22, no. 1, pp. 360-6.). T1 images were then spatially normalized to the MNI152 template in Montreal Neurological Institute (MNI) space using the computational anatomy toolbox (CAT) (Gaser, C, Dahnke, R, Thompson, PM, Kurth, F, Luders, E & The Alzheimer's Disease Neuroimaging, I (2024), 'CAT: a computational anatomy toolbox for the analysis of structural MRI data', Gigascience, vol. 13.).<br><br>For both the c-Fos density and the beta-values, a correlation matrix was generated for each group using GraphPad Prism 9.3.1. Adjacency matrices were created using Microsoft Excel. For graphing of networks, Gephi Software v0.10.1 was used. Specific parameters are described in the manuscript. Estimation statistics were computed using the web application built by Hung Nguyen (estimationstats.com), which utilizes the Python code developed by (Ho, J, Tumkaya, T, Aryal, S, Choi, H & Claridge-Chang, A (2019), 'Moving beyond P values: data analysis with estimation graphics', Nat Methods, vol. 16, no. 7, pp. 565-566.).

For manuscripts utilizing custom algorithms or software that are central to the research but not yet described in published literature, software must be made available to editors and reviewers. We strongly encourage code deposition in a community repository (e.g. GitHub). See the Nature Portfolio guidelines for submitting code & software for further information.

Data

Policy information about availability of data

All manuscripts must include a data availability statement. This statement should provide the following information, where applicable:

- Accession codes, unique identifiers, or web links for publicly available datasets
- A description of any restrictions on data availability
- For clinical datasets or third party data, please ensure that the statement adheres to our policy

All source data for all graphs and Figures in the paper can be found in the Supplementary Data. All other data are available from the corresponding author upon reasonable request. This includes raw and processed imaging data, behavioural datasets, and analysis scripts used in this study. Due to ethical considerations, raw human data have been de-identified to ensure privacy and confidentiality in compliance with applicable guidelines and regulations. Access to these data may require approval from the appropriate ethics board and completion of a data-sharing agreement. Animal data, including c-Fos expression maps and functional connectivity matrices, as well as the custom analysis code, are available directly from the corresponding author upon request.

Research involving human participants, their data, or biological material

Policy information about studies with human participants or human data. See also policy information about sex, gender (identity/presentation), and sexual orientation and race, ethnicity and racism.

Reporting on sex and gender	We confirm that the terms 'sex' and 'gender' are used appropriately. We have indicated in the manuscript that for rat experiments only male rats were used, while for human experiments both genders were included. For human experiments gender was determined based on self-reporting. Forty-seven healthy control participants were recruited for the study (25 male, 22 female; mean age, 24.0±0.5 years [± SEM]; range 19–37 years)
Reporting on race, ethnicity, or other socially relevant groupings	The race and ethnicity (or other socially relevant grouping) of human participants was not a variable of interest for this study.
Population characteristics	See above
Recruitment	Participants were recruited through experimental notices distributed throughout the university of Melbourne, Australia. Due to the deceptive nature of the experiment (placebo analgesia and nocebo hyperalgesia), they were informed this study was investigating "human brain imaging of pain", looking at neural responses after application of a neutral control and active creams.
Ethics oversight	All experimental procedures were approved by the University of Sydney Human Research Ethics Committee (HREC:2019/037) and were consistent with the Declaration of Helsinki. Animal experimental protocols used in this study were approved by the University of Sydney Animal Care and Ethics Committee (Project Number 1165). All procedures followed the guidelines outlined by the NHMRC's 'Code for the Care and Use of Animals in Research' and the NSW Animal Research Act (2007). The principles of the three R's (replacement, reduction, and refinement) were strictly applied to minimize pain and discomfort, and the study adhered to the IASP's 'Ethical Guidelines for Investigations of Experimental Pain in Conscious Animals.'

Note that full information on the approval of the study protocol must also be provided in the manuscript.

Field-specific reporting

Please select the one below that is the best fit for your research. If you are not sure, read the appropriate sections before making your selection.

☐ Life sciences ☒ Behavioural & social sciences ☐ Ecological, evolutionary & environmental sciences

Behavioural & social sciences study design

All studies must disclose on these points even when the disclosure is negative.

Study description	This study involved conditioning healthy human participants to believe a sham placebo cream labelled and described as "lidocaine" (a potent analgesic) and sham nocebo cream labelled "capsaicin" (a potent hyperalgesic) were working to modulate their pain relative to a control vaseline cream. fMRI was then used to assess brain activity during these responses.
Research sample	The research sample was largely formed by University students and researchers in adjacent fields at the University of Melbourne.
Sampling strategy	An apriori power analysis was conducted using Eippert et al. (2009) findings of cortico-brainstem communication. This revealed a total sample size of at least 40 would be necessary to detect similar effect sizes with 95% power (d = 0.31, α = 0.05, power = 0.95). We elected to sample a larger number of participants due to signal artefact which can arise during human brain imaging, excluding some functional datasets. We observed no significant signal or structural artefact in any of our 47 participants, and as such included all functional data to meet the criteria of our power analysis and bolster the strength of any potential findings.
Data collection	Human brain imaging was recorded using the 7T MRI described above. Noxious stimuli were applied using a 3x3cm Peltier element thermode (Medoc). Pain rating data was recorded throughout the course of the study using a Visual Analogue Scale, which participants used to dynamically report their pain both outside (conditioning and reinforcement) and inside (test) the scanner.
Timing	Data collection occurred consistently throughout the years 2021-2022. Due to the constraints induced by COVID-19, we were unable to collect any data throughout lockdown periods in Australia.
Data exclusions	No data was excluded from this study
Non-participation	No participant drop out occurred throughout this study.
Randomization	Participants were allocated to a placebo "responder" or "nonresponder" group using a bootstrapped permutation procedure for determining significant deviations in typical pain processing that occur during placebo analgesia and nocebo hyperalgesia. This method involves mean VAS ratings to each of the 8 noxious stimuli delivered during the control stimulated series being entered to a permutation model, where 10,000 artificial sample are generated with replacement. This artificial sample is then significance tested to 10,000 artificial samples generated from the VAS ratings to each 8 noxious stimuli delivered during the 'lidocaine' stimulated series. If the mean difference between the two series was significant, with the 'lidocaine' significantly lower than the control, a participant was considered a responder. If not, they were considered a non-responder. The vise versa is true for nocebo hyperalgesia, where if a significant increase is present during the 'capsaicin' stimulated series, participants are considered nocebo responders.

Reporting for specific materials, systems and methods

We require information from authors about some types of materials, experimental systems and methods used in many studies. Here, indicate whether each material, system or method listed is relevant to your study. If you are not sure if a list item applies to your research, read the appropriate section before selecting a response.

Materials & experimental systems

n/a	Involved in the study	
☐	☒	Antibodies
☒	☐	Eukaryotic cell lines
☒	☐	Palaeontology and archaeology
☐	☒	Animals and other organisms
☒	☐	Clinical data
☒	☐	Dual use research of concern
☒	☐	Plants

Methods

n/a	Involved in the study	
☒	☐	ChIP-seq
☒	☐	Flow cytometry
☐	☒	MRI-based neuroimaging

Antibodies

Antibodies used	Primary c-Fos antibody was obtained from Abcam RRID: AB_2737414. Secondary antibody was obtained from Vector Labs: RRID: AB_2336201
Validation	Abcam RRID: AB_2737414 (c-Fos 190289) has been validated by the manufacturer, has been used in 216 publications to date. See manufacturer website for further validation information.<br><br>Similarly, Vector Labs: RRID: AB_2336201 (BA1100) has been used in over 1000 citations and has been validated in fixed frozen rat tissue as was used in the study. See manufacturer website for further validation information.

Animals and other research organisms

Policy information about studies involving animals; ARRIVE guidelines recommended for reporting animal research, and Sex and Gender in Research

Laboratory animals	Six-week-old male (n=50) Sprague-Dawley rats (ARC, Perth, WA, Australia), weighing 170-220g upon arrival were used for these experiments.
Wild animals	Study did not involve wild animals
Reporting on sex	This study used only male rats as a previous study from our lab systematically investigated sex as a variable for placebo responses in rats and found that there was no significant sex difference in the ability to produce placebo responses (Boorman and Keay 2021 - 'Morphine-conditioned placebo analgesia in female and male rats with chronic neuropathic pain: c-Fos expression in the rostral ventromedial medulla').
Field-collected samples	Study did not involve field collected samples
Ethics oversight	The experimental protocols used in this study were approved by the University of Sydney Animal Care and Ethics Committee (Project Number 1165). All procedures followed the guidelines outlined by the NHMRC's 'Code for the Care and Use of Animals in Research' and the NSW Animal Research Act (2007). The principles of the three R's (replacement, reduction, and refinement) were strictly applied to minimize pain and discomfort, and the study adhered to the IASP's 'Ethical Guidelines for Investigations of Experimental Pain in Conscious Animals.'

Note that full information on the approval of the study protocol must also be provided in the manuscript.

Plants

Seed stocks	No seeds used
Novel plant genotypes	No plants used
Authentication	*Describe any authentication procedures for each seed stock used or novel genotype generated. Describe any experiments used to assess the effect of a mutation and, where applicable, how potential secondary effects (e.g. second site T-DNA insertions, mosiacism, off-target gene editing) were examined.*

Magnetic resonance imaging

Experimental design

Design type	Event-related design
Design specifications	Eight acute noxious stimuli were applied across the course of each functional sequence. Each stimulus lasted a total of 15 seconds, including a 4 degree / second ramp up from and ramp down to baseline.
Behavioral performance measures	Ongoing pain responses were recorded throughout all experimental phases using a computerized Visual Analogue Scale (VAS). That is, this rating system dynamically recorded participant pain responses at all times on a time scale consistent with volumes recorded during fMRI collection.

Acquisition

Imaging type(s)	Structural (T1-weighted), and functional MRI (fMRI)
Field strength	7-Tesla
Sequence & imaging parameters	A T1-weighted anatomical image set covering the whole brain was collected (repetition time=5000 ms, echo time=3.1ms, raw voxel size=0.73x0.73x0.73mm, 224 sagittal slices, scan time=7mins). The two fMRI acquisitions (either control and 'lidocaine' placebo or 'capsaicin' nocebo) each consisted of a series of 134 gradient echo echo-planar measurements using blood oxygen level dependant (BOLD) contrast covering the entire brain. Images were acquired in an interleaved collection pattern with a multi-band factor of four and an acceleration factor of three (repetition time=2500ms, echo time=26ms; raw voxel size=1.0x1.0x1.2mm, 124 axial slices, scan time=6:25mins).
Area of acquisition	Whole brain coverage was recorded in both the T1-weighted and Functional brain scans

Diffusion MRI ☐ Used ☒ Not used

Preprocessing

Preprocessing software

Statistical Parametric Mapping Version 12 (SPM12) and the spatially unbiased infratentorial template (SUIT) toolbox for brainstem isolation and normalization. The first five volumes of each scan were removed from the model due to excessive signal saturation from the scanner. The remaining 129 functional images were slice-time and motion corrected and the resulting 6 directional movement parameters were inspected to ensure that all fMRI scans had no greater than 1mm of linear movement or 0.5 degrees of rotation movement in any direction. Images were spatially smoothed using a 6mm Full-width-at-half-maximum (FWHM) gaussian kernel for wholebrain and 1mm FWHM gaussian for brainstem-isolated.

Normalization

Each individual’s fMRI image sets were then coregistered to their own T1-weighted anatomical, the T1 was then spatially normalized to the MNI152 template in Montreal Neurological Institute (MNI) space and the parameters applied to the fMRI image sets. For brainstem-isolated images, a brainstem-isolated T1 image was generated and normalized to the SUIT template in MNI space. these normalization parameters were then applied to the fMRI series following a personalized masking procedure consistent with the brainstem isolated T1 image.

Normalization template

MNI152 template in MNI space for wholebrain and SUIT for brainstem

Noise and artifact removal

Images were then linearly detrended to remove global signal changes, physiological noise relating to cardiac and respiratory frequency was removed using the DRIFTER toolbox (Särkkä, S. et al. NeuroImage 60, 1517-1527, (2012)), and the 6-parameter movement related signal changes were modelled and removed using a linear modelling of realignment parameters (LMRP) procedure.

Volume censoring

The first five volumes of each scan were removed from the model due to excessive signal saturation from the scanner.

Statistical modeling & inference

Model type and settings

A repeating boxcar model convolved with a hemodynamic delay function (TR=2.5s) was applied to each of the fMRI series to isolate volumes where noxious stimuli were applied to either the control or 'lidocaine' placebo / 'capsaicin' nocebo cream relative to no pain periods.

Effect(s) tested

Main effects of placebo and nocebo were determined by calculating effect size changes in beta-value (coherence in BOLD signal change with the repeating boxcar model) between either the control and 'lidocaine' placebo or control and 'capsaicin' nocebo fMRI series. Main effect of response was determined by calculating identical statistical measures as above however only in those participants that demnstrated significant placebo analgesic or nocebo hyperalgesic responses as determined through permutation testing described previously. These changes were post-hoc compared to nonresponders to ensure this effect was present only in those participants demonstrating significant pain modulatory effects.

Specify type of analysis: ☐ Whole brain ☒ ROI-based ☐ Both

Anatomical location(s)

Pain perceptual and modulatory regions of the cortex and brainstem as a restricted list of the Human Connectome Project Atlas Extended (HCP-ex), with additional brainstem ROI's with a known role in pain processing / modulation. Supplementary table 1 provides an exhaustive list of these ROI's, however, largely these consist of prefrontal, cingulate, parietal and thalamic ROI's in the cortex, and the PAG-RVM system with addition of the locus coeruleus and medullary sites in the brainstem.

Statistic type for inference

(See Eklund et al. 2016)

Cohen's D effect sizes were calculated within each ROI determining overall change in signal intensity between the control and either placebo or nocebo fMRI series.

Correction

As we calculated overall signal change across each apriori defined ROI, no statistical correction in terms of cluster forming threshold or extent threshold was applied. That is, an average measure was extracted from each voxel forming each ROI. Estimation statistics were computed using the web application built by Hung Nguyen (estimationstats.com), which utilizes the Python code developed by (Ho et al., 2019). For each test, effect sizes (Cohen's d) and their associated 95% confidence intervals were calculated using bias-corrected and accelerated bootstrap resampling with replacement, with 5000 bootstrap samples applied per test.

Models & analysis

n/a	Involved in the study
☐	☒ Functional and/or effective connectivity
☐	☒ Graph analysis
☒	☐ Multivariate modeling or predictive analysis

Functional and/or effective connectivity

Correlation matrices were generated printing R-values of linear regression between each of the ROI's included in this investigation. Specifically, in each of the 47 human participants, their mean BOLD value in one ROI was linearly regressed against their own value in the other ROI's and this process repeated until an entire matrix had formed demonstrating how closely overall activity in one cortical / brainstem site was consistent with change in another site.

Graph analysis

To visualize the functional connectivity graphs for both cfos densities (rats) and beta values (human), we used Gephi software v0.10.1. The adjacency matrices, which were calculated from the correlation matrices,

were imported into Gephi as undirected connections.

Finally, we pruned these networks by cross-referencing with anatomical data from the Rat Connectome Project, ensuring that only connections with known anatomical pathways remained in the analysis. The connectome provided further details about the strength and direction of each connection, assigning connection strengths ranging from 0.5 (very light) to 4 (very strong), forming the basis of the final neural circuitry model.
Similar to the functional connectivity analysis, adjacency matrices were generated from the correlation matrices and imported into Gephi as directed connections.

We applied the Force Atlas layout with the following parameters: inertia = 0.1, repulsion strength = 1000, attraction strength = 10, maximum displacement = 1000, auto stabilize function = on, autostab strength = 80, autostab sensibility = 0.2, gravity = 3000, attraction distribution = off, adjust by sizes = on, and speed = 1.0. Node size was determined by the degree of connectivity, where more connected nodes appeared larger, while the size of the arrows represented the strength of the anatomical projection.
